# Emulation of Biological Synapse Characteristics from Cu/AlN/TiN Conductive Bridge Random Access Memory

**DOI:** 10.3390/nano10091709

**Published:** 2020-08-29

**Authors:** Hyojong Cho, Sungjun Kim

**Affiliations:** Division of Electronics and Electrical Engineering, Dongguk University, Seoul 04620, Korea; chj9102@dgu.ac.kr

**Keywords:** CBRAM, metal nitride, resistive switching, synaptic device, neuromorphic

## Abstract

Here, we present the synaptic characteristics of AlN-based conductive bridge random access memory (CBRAM) as a synaptic device for neuromorphic systems. Both non-volatile and volatile memory are observed by simply controlling the strength of the Cu filament inside the AlN film. For non-volatile switching induced by high compliance current (CC), good retention with a strong Cu metallic filament is verified. Low-resistance state (LRS) and high-resistance state (HRS) conduction follow metallic Ohmic and trap-assisted tunneling (TAT), respectively, which are supported by I–V fitting and temperature dependence. The transition from long-term plasticity (LTP) to short-term plasticity (STP) is demonstrated by increasing the pulse interval time for synaptic device application. Also, paired-pulse facilitation (PPF) in the nervous system is mimicked by sending two identical pulses to the CBRAM device to induce STP. Finally, potentiation and depression are achieved by gradually increasing the set and reset voltage in pulse transient mode.

## 1. Introduction

Neuromorphic systems are attracting attention as replacements for von Neumann architecture in the near future due to their capacity for energy efficient, massively parallel, and error tolerant computing [1,2,3,4]. Two-terminal resistive switching memory possesses many features that are suitable for synaptic devices for neuromorphic systems, such as low-power operation, high scalability, analogue switching for multi-level cells (MLCs), fast switching, high endurance, and long retention [5,6,7,8,9,10]. The various resistive switching behaviors have been observed from a lot of materials such as oxides, nitrides, and chalcogenides [11,12].

A CBRAM device is a type of resistive switching memory that involves diffusion of highly diffusive metal electrodes such as Ag and Cu into a host insulator to form a metallic conducting filament in a manner that depends on applied voltage [13,14,15,16]. Ion movement in a CBRAM generally allows for faster operation speeds and lower voltage levels than are seen in filamentary and non-filamentary bipolar devices [17,18]. In terms of metal selection for the top CBRAM electrode, Cu is superior to Ag in terms of CMOS-friendly processes when using a barrier metals such as TaN [19] and TiN [20]. Metal oxides such as HfO_2_ and Al_2_O_3_ are popular host insulators in CBRAM devices owing to their stable and reliable resistive switching characteristics compared to organic materials. Recently, nitride-based resistive switching compounds, such as AlN, ZrN, HfN, NiN, and SiN, have been reported to have excellent non-volatile properties comparable to metal oxides in terms of operation speed, endurance, and retention [21,22,23,24,25,26,27,28]. Especially, AlN is suitable for resistive switching memory owing to its high thermal conductivity and large band gap with good insulating properties [29,30,31,32] and could be improved by additional approaches such as the scaling, bilayer structure, and doping like oxide-based RRAM. Robust resistive switching was reported for the TiN/AlN/TiN device [33] and Pt/AlN:Cu/PT device [34] for non-volatile memory applications. However, there have been few studies on volatile and non-volatile switching for selector and synaptic device applications in the neuromorphic system.

In this work, we fabricated a CBRAM-type AlN-based metal-insulator-metal (MIM) device and investigated the STP and LTP for a neuromorphic system. The coexistence of non-volatile and volatile switching is demonstrated by controlling the CC in DC sweep mode. STP is well-emulated by varying pulse interval time in pulse responses. Also, PPF is well-mimicked by changing the interval time and decay rate for STP. The conduction mechanisms of LRS and HRS are explained by I–V fitting and temperature dependence. Finally, we demonstrated 10 cycles of potentiation and depression with incremental pulse schemes.

## 2. Materials and Methods

The Cu/AlN/TiN device was fabricated in the following manner. A 100 nm thick TiN layer was prepared on a SiO_2_/Si wafer using a sputtering system. A 20 nm thick AlN layer was deposited by sputtering. The Al target was sputtered with N_2_ (12 sccm) and Ar (8 sccm) gases under a sputtering power of 0.5 kW, pulsed DC, and 50 kHz, and a pressure of 3 mTorr. A 100-nm thick Cu top electrode was deposited by e-beam evaporation and patterned by a shadow mask containing circular patterns with a diameter of 100 μm. The electrical properties in the DC sweep and transient modes were measured using a semiconductor parameter analyzer (Keithley 4200-SCS and 4225-PMU ultrafast module, Solon, OH, USA). During the measurements, a bias voltage and pulse were applied to the Cu top electrode while the TiN bottom electrode was grounded. XPS depth analysis was conducted with a Nexsa (ThermoFisher Scientific, Waltham, MA, USA) with a Microfocus monochromatic X-ray source (Al-Kα (1486.6 eV), a sputter source (Ar^+^), an ion energy of 2 KV, and a beam size of 100 μm.

## 3. Results and Discussion

**Figure 1:**
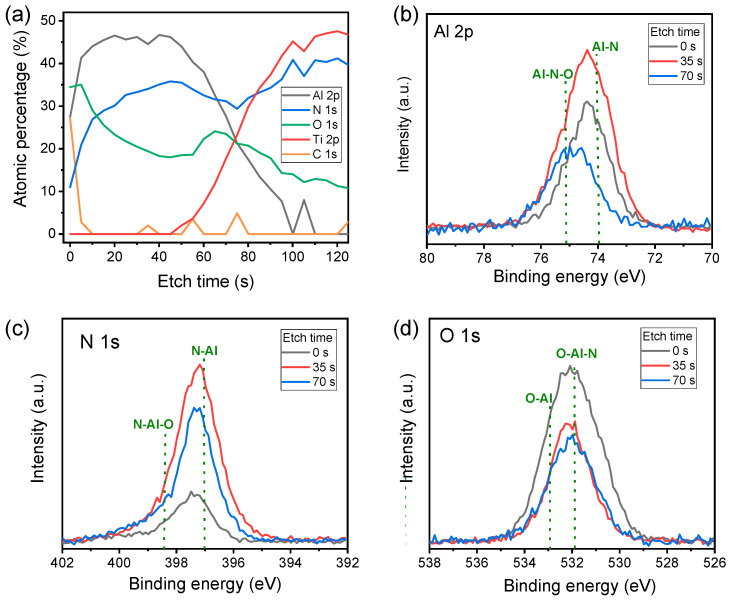
XPS analysis of AlN/TiN layers: (**a**) Depth profiles for elements Al, N, O, Ti, and C; (**b**) Al 2p, (**c**) N 1s; (**d**) O 1s spectra.

Figure 1a shows the XPS depth profile of the AlN/TiN layers for the elements Al, N, O, Ti, and C. The oxygen content is high and the Al content is low at the beginning of etching because oxygen always exists on a sample exposed to the atmosphere. It is also difficult to completely exclude reactions with a little oxygen during AlN deposition and Al target is easily oxidized. At the beginning of AlN deposition, the oxygen in the chamber reacts first, and the oxygen concentration increases with AlN depth. We also confirm that the gradient of oxygen concentration from the peak shifts left in the Al 2p spectra from 35 s to 70 s because the peak for Al–N–O bonds has a higher binding energy (75.1 eV) than that of Al–N (73.9 eV) (Figure 1b) [35,36]. Also, TiN bottom electrode could be little oxidized before AlN deposition. The intensity of Al 2p is highest at 35 s because atmospheric absorption of O–H increases at 0 s and Ti content relatively increases at 70 s. The intensities of the N 1s and O 1s spectra as a function of binding energy with different etching times are presented in Figure 1c and d. The peak slightly shifts left from 35 s to 70 s in the N 1s spectra because the amount of N–Al–O bonds (398.4 eV) is increased at the interface between the AlN and TiN layers.

**Figure 2:**
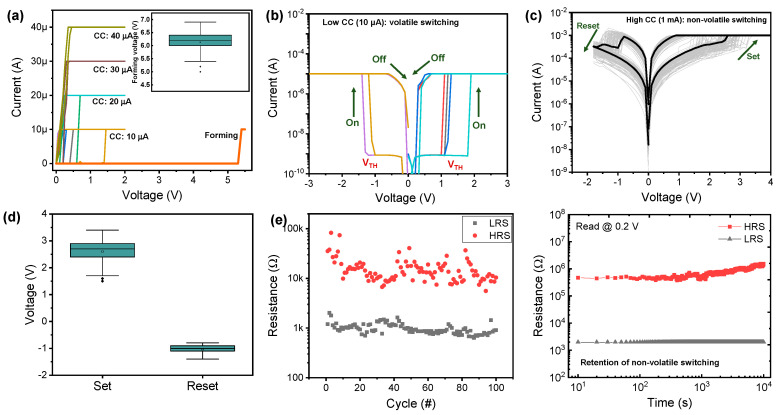
Volatile and non-volatile switching of Cu/AlN/TiN device. (**a**) Voltage sweep with different CCs from 10 μA to 40 μA to distinguish the volatile and non-volatile switching and the distribution of forming voltage in inset; (**b**) Volatile I–V and (**c**) non-volatile I–V characteristics; (**d**) Set and reset distribution and (**e**) endurance for non-volatile switching; (**f**) Retention property of non-volatile switching in the LRS and HRS for 10,000 s.

Firstly, the volatile switching and non-volatile switching can be distinguished by CC. Figure 2a shows the I–V curves with different CCs from 10 μA to 40 μA. The volatile switching is observed with CC of 10 μA and 20 μA and the non-volatile switching is observed with higher CCs of 30 μA and 40 μA. For volatile switching and nonvolatile switching, the initial current and high LRS current are observed. Figure 2b shows the I–V characteristics of volatile switching in the Cu/AlN/TiN device. In the initial state, the current rises rapidly with a steep slope at the threshold voltage (V_TH_). Here, a low CC (10 μA) is required to achieve volatile switching with a small Cu conducting filament. After the device turns on, it seems to maintain some current in the back sweep, but when it repeatedly performs with the same voltage sweep, it turns on again from the initial state, indicating volatile switching. Bidirectional volatile switching is suitable for a selector device that can suppress the current in the voltage region less than V_TH_ and provide enough current in the voltage region higher than V_TH_ for the set and reset processes. On the other hand, the non-volatile memory property is observed at a higher CC (1 mA) in the same device. The set and reset processes occur via positive sweep and negative sweep, respectively, which is typical bipolar resistive switching behavior (Figure 2c). The set process changes the device from a high-resistance state (HRS) to a low-resistance state (LRS); the reset process is the opposite of the set process and returns the device to a HRS, indicating typical filamentary-type bipolar switching. The average values of set voltage and reset voltage are 2.602 V and −1.033 V and the relative deviations of set voltage and reset voltage are 0.167 V and 0.137 V (Figure 2d). Moreover, the endurance cycles of 100 are achieved by set and reset process (Figure 2e). Figure 2e shows good retention in the LRS and HRS. The LRS and HRS resistance values read at 0.2 V do not deteriorate significantly and the distinction between the two states is maintained for 10,000 s. The high CC makes a strong conducting filament, and this filament cannot be changed within a certain period of time while maintaining the non-volatile property.

**Figure 3:**
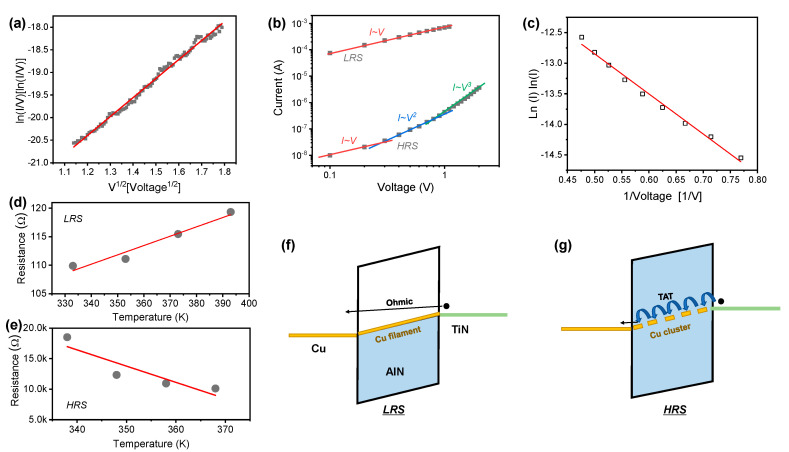
Conduction mechanism of Cu/AlN/TiN device. (**a**) ln(*I*/*V*) versus *V*^1/2^ for Poole-Frenkel emission; (**b**) Log-log fitting of non-volatile *I*–*V* curves for power law; (**c**) ln(I) versus 1/*V* for TAT model; Temperature dependence in the (**d**) LRS and (**e**) HRS; (**f**) Ohmic and (**g**) TAT model in band diagrams in the LRS and HRS, respectively.

Next, we investigate the conduction mechanism of the Cu/AlN/TiN device. Figure 3a shows ln(*I*/*V*) versus *V*^1/2^ of the initial current before the forming. The linear fitting indicates that the carrier transport of the pristine device follows bulk-limited Poole-Frenkel emission [37]. AlN film has nitride-related defects that acts as the trap sites of the electron. Therefore, the emission of trapped electron into the conduction band could occur [26]. We created double logarithmic plots of *I*–*V* curves in the LRS and HRS (Figure 3b). The *I*–*V* curve in the LRS is well-fitted with slope 1, which follows Ohmic behavior. The resistance in the LRS increases with temperature, indicating that a Cu conducting filament with metallic behavior has formed in the AlN film in Figure 3d. On the other hand, there are three distinctive regimes with different slopes in the HRS, which is explained by the SCLC model [38,39]. Thermally generated carriers are dominant over electrode-injected carriers in the Ohmic conduction regime (*I*–*V*) at low voltage. The slope increases when the injected carriers become dominant over the thermally generated carriers in a higher voltage regime (*I*–*V^2^*). The slope increases in the third regime (*I*–*V^3^*) when the traps in AlN are all filled out by carriers due to the further increase in the electric field. Resistance decreases with increasing temperature in the HRS, as shown in Figure 3e. This suggests that the Cu filament does not dominate conduction in the HRS because the Cu filament has ruptured. Figure 3c shows ln(*I*) versus 1/*V* for TAT at the voltage region (1.3 V~2.1 V) in the HRS [40,41]. The tunneling emission of the electrons helped by the defects in the dielectric can be accelerated in high electric field. Cu cluster may remain in the HRS because Cu filament is not fully ruptured considering that HRS current is higher than initial current. The electron can move via multiple trap sites that Cu clusters provide (Figure 3g). On the other hand, in the LRS case, there is no barrier seen from TiN bottom electrode since the strong Cu filament are formed in the AlN layer. Therefore, metallic Ohmic conduction would be dominant (Figure 3f).

**Figure 4:**
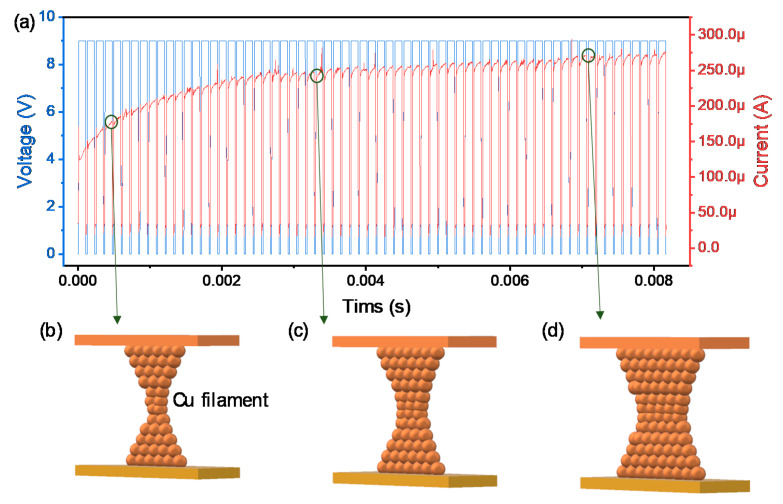
Transient characteristics of Cu/AlN/TiN device for (**a**) LTP; Schematics of Cu filament growing (**b**–**d**).

Next, the transient characteristics given pulse responses are investigated in the Cu/AlN/TiN device for neuromorphic applications. Here we employ the initial current for volatile switching properties to distinguish the non-volatile and volatile switching by pulse width and interval time. When a pulse train with an amplitude of 9 V is applied to the device in the initial state, the current gradually increases because of the property of LTP (Figure 4a). This behavior is similar to the long-term memory of some information in the human brain when a stimulus is biologically applied. Here, the filament size, once it is formed, can be expected to gradually increase without decomposition due to the continuous pulse inputs (Figure 4b–d).

**Figure 5:**
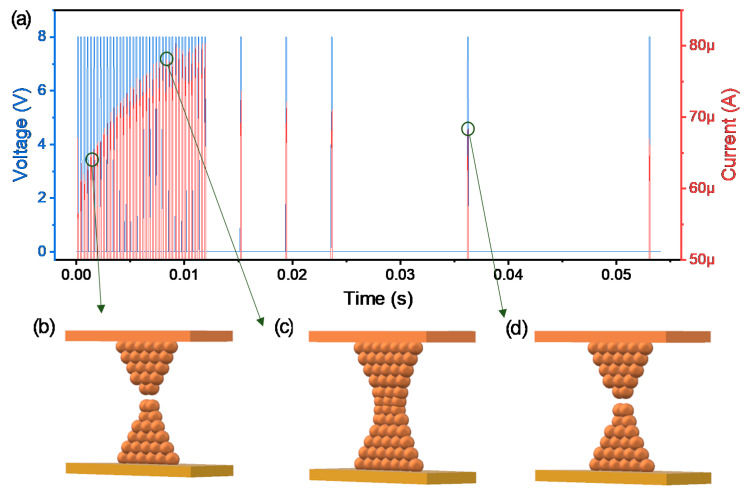
Transient characteristics of Cu/AlN/TiN device for (**a**) STP; Schematics of Cu filament (**b**) Weak filament before evolution; (**c**) growing; (**d**) Spontaneous decomposition.

On the other hand, when a pulse train with an amplitude of 8 V is applied at a similar frequency, an increase in current is observed, and the increased current gradually decreases when the pulse frequency is decreased (Figure 5a). Here, the smaller voltage amplitude is a precondition for weakening of the conducting filament compared to the voltage seen in Figure 4a, which can be verified by monitoring at the value of the transient current. Here, the phenomenon of gradual current decrease when the frequency of stimulation decreases can be likened to STM in the human brain. The gradual attenuation of the current indicates a process in which the size of the weakly formed filament spontaneously decomposes when the frequency of input stimulation is reduced (Figure 5b,c).

**Figure 6:**
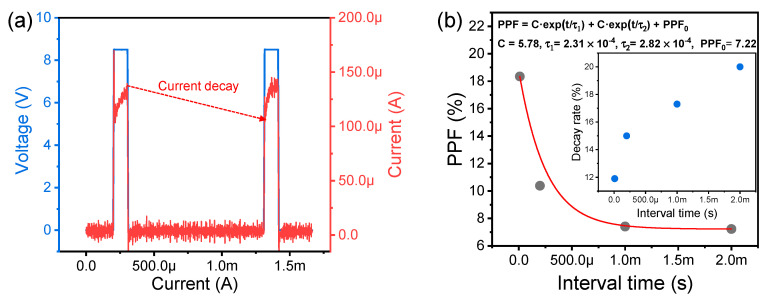
PPF characteristics of the Cu/AlN/TiN device: (**a**) Transient characteristics given two identical pulse inputs; (**b**) PPF and decay rate as a function of interval time.

Next, we mimic PPF, which is a biological synaptic activity related to STP and activity-dependent synaptic plasticity [42]. STM can be used for reservoir computing, such as for temporal data processing with low training costs. PPF is emulated when two identical pulse inputs (amplitude: 8.5 V and width: 100 μA) are applied to the Cu/AlN/TiN device. The current increases temporarily at the end of the first pulse and then decreases at the beginning of the second pulse. As the interval time increases, the rate of current decay increases, which is related to STP (Figure 6a, inset). This suggests that the conducting filament formed by the first pulse input instantaneously shrinks. Figure 6b shows PPF as a function of the time interval between the two pulses. The PPF value is defined as ((*I*_1st_ *− I*_2nd_)/*I*_1st_) × 100% where *I*_1st_ is the current at the midpoint of the first pulse input and I_2nd_ is the current at the midpoint of the second pulse input. Here, the current during the rise and fall of the pulse input is influenced by the displacement current, so the current is sensed at the center of the pulse input to obtain the exact PPF value. PPF exponentially decreases and the decrease is well fitted with the following Equation (1):

C·exp(−t/τ_1_) + C·exp(−t/τ_2_) + PPF_0_ (1)

where C is fitting constants, τ_1_ and τ_2_ are time constants, and PPF_0_ is the saturated PPF. Here, PPF_0_ is not zero with a very long interval, which means that the Cu filament formed under the two pulse inputs does not completely decompose. Therefore, this result is closer to a soft set process rather than pure volatile switching.

**Figure 7:**
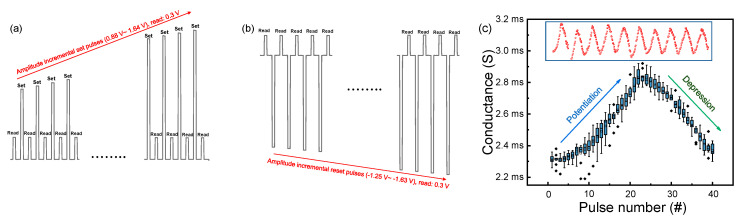
LTP and LTD characteristics of the Cu/AlN/TiN device. (**a**) Incremental pulse schemes for (**a**) potentiation and (**b**) depression; (**c**) LTP and LTD characteristics in 10 cycles.

Next, we implement LTP and LTD of the Cu/AlN/TiN device using incremental amplitude pulse schemes. After the forming process, the high current region is employed for non-volatile potentiation and depression, which is well matched with DC sweep in Figure 2c. Non-volatile resistive switching in the Cu/AlN/TiN device follows a filamentary model, indicating that it is difficult to obtain MLC with fine adjustments. Therefore, incremental amplitude pulse responses are employed to obtain multiple conductance levels. The set pulse with a width of 1 ms is increased from 0.88 V to 1.64 V and a read voltage of 0.3 V is inserted between the set pulses to extract the conductance seen in Figure 7a. Similarly, the reset pulse with a width of 1 ms is increased from −1.25 V to −1.63 V and a read voltage of 0.3 V is used in Figure 7b. With 20 consecutive set pulses followed by 20 consecutive reset pulses, 10 cycles of potentiation and depression are achieved (Figure 7c, inset). Figure 6c shows the average value and spread of the 10 cycles, where the variations in potentiation and depression are 52.3 μS and 41.6 μS, respectively. Cycle-to-cycle variation as well as the linear weight update method are important to achievement of accurate learning in a neuromorphic system.

Finally, we compare this work with existing literature of nitride based CBRAM in terms of deposition method of the dielectrics, operation voltage and current, endurance cycle, retention, and the applications in Table 1. The most deposition techniques were prepared by DC reactive sputtering or RF sputtering for good resistive switching. Ag electrode tends to smaller operation voltage compared to the Cu electrode due to high diffusivity [21,26,43,44,45]. The selector and synaptic device applications are demonstrated even though the resistive switching performances of Cu/AlN/TiN in this work are not overwhelming compared to the previous works.

## 4. Conclusions

In summary, we closely investigated resistive switching and the synaptic characteristics of a Cu/AlN/TiN device. Volatile and non-volatile properties are observed by controlling the CC under DC sweep. The Ohmic conduction and TAT are revealed by the fitting process and temperature dependence for non-volatile resistive switching. Moreover, various pulse schemes are used to trace the synaptic dynamics of STM and LTM. The transition from LTP to STP is achieved by reducing the pulse amplitude and interval time. Also, PPF is emulated by varying the pulse interval time for STP and the facilitation effect. Finally, repeated LTP and LTD characteristics are statistically obtained at high current levels.

## Figures and Tables

**Table 1:** Comparison of the resistive switching performances among nitride-based CBRAM devices.

Device Structure	Dielectric Deposition Method	Operation Voltage	Operation Current	Endurance Cycle	Retention	Applications
Cu/AlN/Pt [21]	DC sputtering	Set: ~3 V Reset: ~−2 V	>1 mA	~1000	10^6^ s at RT	Non-volatile memory
Ag/AlN/Pt [26]	RF magnetron sputtering	Set: ~0.5 V Reset: ~−0.5 V	>100 μA	-	-	Non-volatile memory
Cu/AlN/Pt [43]	DC reactive magnetron sputtering	Set: ~3 V Reset: ~−2 V	>10 mA	~10,000	10^4^ s at RT	Non-volatile memory
Pt/AlN/Cu/AlN/Pt [44]	RF magnetron sputtering	~±3 V	<100 μA	~50	12,000 s at RT	Non-volatile memory
Pt/AlN/Ag/AlN/Pt [44]	RF magnetron sputtering	~±2 V	<100 μA	~500	12,000 s at RT	Non-volatile memory
Ag/a-BN/Pt [45]	RF magnetron sputtering	Set: ~1 V Reset: ~−1 V	<1 mA	~1000	12,000 s at 85 °C	Non-volatile memory
Cu/AlN/TiN [this work]	DC pulsed sputtering	Set: ~3.5 V Reset: ~−1.5 V	>1 mA	100	10,000 s RT	Non-volatile memory, Selector device, Synaptic device
